# Development and validation of a motivation scale for health behavior change in patients with coronary heart disease: a cross-sectional study

**DOI:** 10.1186/s40359-026-05114-z

**Published:** 2026-07-03

**Authors:** Yating Lu, Ying Wu, Meihua Ji, Ying Deng, Lixin Zhang, Yuling Chen, Yiqiang Guo

**Affiliations:** 1 School of Nursing, Capital Medical University, 10 You-an-men Wai Xi-tou- tiao, Feng-tai District, Beijing, 100069 China; 2 School of Nursing, Beijing University of Chinese Medicine, Beijing, China; 3 Ward 2, Coronary Heart Disease Center, Department of Cardiology, Beijing Anzhen Hospital, Capital Medical University, Beijing, China; 4 School of Nursing, Johns Hopkins University, Maryland, USA; 5 Nursing and Health Education Research Centre, Kiang Wu Nursing College of Macau, Macau SAR, China

**Keywords:** Coronary Heart Disease, Health Behavior Change, Motivation, Psychological Assessment, Psychometric Validation

## Abstract

**Background:**

Motivation is a critical psychological determinant of health behavior change in patients with coronary heart disease (CHD). Despite its theoretical and clinical importance, well-validated psychometric instruments specifically designed to assess motivation intensity in this population remain limited. This study aimed to develop and validate a motivation scale for health behavior change in patients with CHD.

**Methods:**

A cross-sectional study was conducted in a tertiary hospital in China between April 2019 and December 2022 using a two-phase scale development design. The conceptual framework was based on the Contemplation-Action-Maintenance model. An initial item pool was generated through a literature review, existing instruments, and expert input, followed by Delphi consultation and patient-based pretesting in 76 patients with CHD. Item selection was performed combining Classical Test Theory and Item Response Theory in 749 patients. Psychometric properties were evaluated in an independent sample of 347 patients, including 35 who completed the scale twice. Reliability and validity were assessed using internal consistency, test–retest reliability, content validity, criterion validity, concurrent validity, and confirmatory factor analysis.

**Results:**

The final scale comprised 12 items across three dimensions, explaining 86.04% of the total variance. The scale demonstrated good internal consistency (Cronbach’s α = 0.906) and test–retest reliability (ICC = 0.981, 95% CI [0.962, 0.990]). Criterion validity was supported by correlations with the General Motivation Subscale and Visual Analog Scale (*r* = 0.469–0.751), while concurrent validity was supported by correlations with the Self-Efficacy Scale and Outcome Expectation Scale (*r* = 0.505–0.595). Confirmatory factor analysis indicated an acceptable model fit.

**Conclusions:**

The Motivation Scale for Health Behavior Change is a brief instrument that demonstrated acceptable reliability and validity in assessing motivation in patients with CHD. It may serve as a useful tool for assessing motivation and informing tailored behavior change interventions.

**Supplementary Information:**

The online version contains supplementary material available at https://doi.org/10.1186/s40359-026-05114-z.

## Background

As a central determinant in behavior change theories, motivation is widely recognized as a key driver of goal-directed behaviors [1, 2]. This is particularly critical for patients with coronary heart disease (CHD), for whom sustained motivation is essential for adopting lifestyle changes such as regular physical activity and healthy eating [3], which are key components of secondary prevention [4]. However, adoption of these behaviors remains suboptimal in this population [5, 6].

In patients with CHD, motivation for health behavior change is shaped by a range of condition-specific psychological and clinical factors. For example, acute cardiac events can act as powerful triggers that temporarily enhance motivation for behavior change [7]. At the same time, fear of recurrent cardiac events, heightened perceived vulnerability, and exercise-related anxiety may hinder motivation for engaging in recommended behaviors [8, 9]. As a result, motivation in this population is dynamic and heterogeneous, varying across individuals and fluctuating over time depending on disease experience, symptom burden, and psychosocial context [10]. These characteristics make it difficult to accurately identify patients’ motivational status using simple or generic approaches and may lead to mismatched or less effective interventions. This highlights the need for more precise and context-sensitive assessment of motivation in patients with CHD.

A review of existing instruments, however, reveals significant limitations for this purpose. Commonly used tools for measuring motivation in health contexts may not be well suited to behavioral adoption in patients with CHD. Visual analog scales (VAS) [11], although simple, lack sensitivity to capture the multifaceted nature of motivation. Scales that conceptualize motivation as the sum of multiple reasons (e.g., Zach’s Physical Activity and Leisure Motivation Scale [12]) typically employ additive scoring. This approach may be inadequate for the CHD population, where a single, powerful health threat can outweigh multiple moderate reasons. Theory-driven scales, such as the Self-Determination Scale [13, 14], focus on motivational quality rather than intensity. Some instruments (e.g., the Motivation to Change Lifestyle and Health Behaviors for Dementia Risk Reduction Scale [15]) assess motivational correlates such as risk perception, rather than motivation itself.

A more nuanced approach is exemplified by Xu’s health behavior motivation scales [16], which attempt to operationalize motivational strength through observable preparatory behaviors, such as willingness to change and planning. However, two key limitations undermine its applicability to the present context. First, at the operational level, its items rely on generic behavioral declarations (e.g., “tend to,” “plan to”) rather than being anchored in specific, contextually relevant preparatory actions. This reduces its sensitivity to capture gradations in motivational strength that are tied to concrete behavioral steps. Second and more fundamentally, its theoretical framework lacks stage specificity. It conflates motivation (the desire and decision to act) with volition (the self-regulatory capacity to maintain action) by including dimensions like “Persistence Motivation,” which measures maintenance self-efficacy (e.g., “I can persist……”), thereby failing to isolate the core construct of interest. These limitations highlight the need for a stage-specific theoretical framework that can distinguish motivational formation from behavior maintenance.

The Contemplation-Action-Maintenance (CAM) model conceptualizes health behavior change as a staged process involving motivational formation (contemplation), action initiation, and maintenance [17]. Unlike broader theories such as Self-Determination Theory, which primarily focus on the quality or type of motivation, the CAM model emphasizes the temporal progression of behavior change and the role of motivation at different stages.

In the present study, the focus is placed on motivation during the contemplation and action phases of the CAM model, rather than across the full continuum of behavior change. Within this stage-specific perspective, motivation intensity is not viewed as a single undifferentiated construct, but as a multidimensional construct reflected in different forms of behavioral readiness. Specifically, it can be manifested in (1) value recognition (the perceived importance of behavior change), (2) planning engagement (the extent to which individuals actively prepare for change), and (3) action initiation (the degree to which individuals begin to implement behavior). These dimensions represent different expressions of the same underlying motivational intensity.

Therefore, this study aimed to develop and validate a novel motivation scale for health behavior change in CHD (MS-HBC). Grounded in the CAM model, the MS-HBC is designed to quantify motivation intensity specifically during the contemplation and action phases. To ensure psychometric rigor, its development integrated both Classical Test Theory (CTT) and Item Response Theory (IRT) for item selection and comprehensive evaluation [18, 19]. This tool may facilitate more precise assessment of motivational processes in patients with CHD and support the development of tailored behavior change interventions.

## Methods

### Study design

This cross-sectional study employed a two-phase scale development process guided by DeVellis’s framework [20]. Phase 1 focused on scale development, including item generation, refinement, and selection to form a preliminary scale. In Phase 2, the scale structure was confirmed, and its reliability and validity were evaluated using an independent sample to ensure psychometric robustness.

## Phase 1. Scale development

### Conceptual Framework

The conceptual framework for the MS-HBC was primarily based on the CAM model, a disease-specific, process-oriented framework for health behavior change in patients with CHD [17]. Within this framework, the scale was designed to assess motivation during the contemplation-to-action transition, with particular emphasis on motivational readiness for initiating health behavior change. Guided by this definition, three core dimensions were derived to represent the motivational continuum leading to action: Health Motivation Tendencies (the foundational desire for health), Planning Motivation (the translation of desire into specific plans), and Action Motivation (the impetus to begin acting). The structure of the scale (e.g., the progression from tendency to plan to action) was also informed by the conceptual progression observed in the first three dimensions of Xu’s health motivation theory [16], providing a complementary, behaviorally-grounded lens for structuring the continuum. The definitions and scope of these three dimensions were subsequently reviewed and confirmed for clarity and relevance by a panel of multidisciplinary experts, ensuring their fit within the CAM framework and the CHD context.

### Item generation and refinement

An initial item pool was generated through a targeted literature review of existing instruments and literature related to health behavior motivation. Electronic databases including PubMed, Web of Science, and CNKI were searched using combinations of keywords such as “motivation,” “health behavior,” and “scale.” Instruments were included if they assessed motivation-related constructs and were published in English or Chinese. A total of 110 items was extracted from 14 instruments (see Additional file 1). Two researchers independently reviewed the items to remove redundancies and ensure relevance, resulting in a 31-item draft.

This draft underwent two rounds of Delphi consultation with 15 multidisciplinary experts from 14 institutions, including psychologists, health management specialists, social scientists, and cardiovascular nursing researchers. Experts rated each item for relevance and clarity on a 5-point Likert scale, and items with an item-level content validity index (I-CVI) below 0.78 were revised or removed based on expert consensus. The process resulted in a revised MS-HBC comprising 18 items across the three pre-defined dimensions.

Subsequently, the draft scale was pilot-tested with 76 patients with CHD (mean age 63.8 ± 12.6 years) to assess item clarity, comprehension, and feasibility. Participants were invited to provide feedback on wording and content coverage following completion of the scale. Feedback was collected using brief semi-structured prompts addressing difficulties in understanding, ambiguous wording, and whether additional behaviors reflecting motivation for health behavior change were not adequately captured by the scale. All feedback was reviewed by two researchers. Minor revisions were made to improve clarity and accessibility of several items. For example, Item 6 was simplified from “If I have undergone surgery or received medication treatment, how important do I think adopting healthy behaviors is?” to “The importance of adopting healthy behaviors after receiving treatment is:”, to improve clarity and align with the statement-based response format used across items. No items were added or removed, and the average completion time for the 18-item scale was 2 min and 25 s.

### Item selection: integrating CTT and IRT

#### Participants and Setting

Participants were recruited by trained research staff during their hospital stay in the cardiology department of a tertiary hospital in Beijing between December 2021 and August 2022. Most participants were clinically stable inpatients receiving routine care or awaiting elective percutaneous coronary intervention. Inclusion criteria were adults with angiography-confirmed CHD; exclusion criteria included diagnosed mental illness, significant sensory impairments, physical disability preventing exercise, or critical/terminal illness.

The sample size was estimated based on multiple considerations. For factor analysis, a subject-to-item ratio of at least 20:1 was recommended [21], resulting in a minimum of 360 participants for the initial 18-item scale. Considering a potential 20% non-response or invalid data rate, the target sample size was at least 450 participants. Additionally, IRT modeling typically requires larger samples, with at least 500 participants recommended to ensure stable parameter estimation [22].

#### Data collection

The initial 18-item MS-HBC is a self-report instrument comprising three subscales. Participants were instructed to consider common lifestyle behaviors relevant to CHD management, including physical activity, diet, smoking cessation, and medication adherence, when responding to the items. To enhance interpretability, participants were encouraged to reflect on the behavior that was most relevant or salient to their current situation when answering the questions.

Items were presented as self-reported descriptions of different aspects of motivation for health behavior change. Participants were asked to rate the extent to which each statement reflected their current situation on a 5-point Likert scale ranging from 1 (extremely low) to 5 (extremely high), indicating the perceived level, frequency, or intensity of the described behavior or attitude. Total and subscale scores were calculated by summing the corresponding item scores, with higher scores indicating greater motivation intensity for health behavior change.

Eligible patients were identified based on medical records and approached in person by the research staff, who explained the study purpose and obtained informed consent. Participation was voluntary, and data collection was arranged at a convenient time for patients to avoid interference with clinical care. Data were collected using paper-and-pencil questionnaires administered in a quiet setting under standardized instructions. Questionnaires were checked immediately upon completion, and any missing responses were clarified at the time of administration to ensure complete datasets.

#### Data analysis

Data were analyzed using SPSS 26.0 (IBM Corp., Armonk, NY, USA) and FlexMIRT 3.6 (Vector Psychometric Group, LLC, Chapel Hill, NC, USA). Demographic characteristics were summarized using frequencies, proportions (%), means, and standard deviations, as appropriate.

Item selection combined CTT and IRT. CTT indices included standard deviation (SD < 0.9), critical ratio (CR < 3), and corrected item-total correlation (CITC < 0.3) with consideration of Cronbach’s α. Exploratory factor analysis (EFA) using principal component analysis, with Kaiser-Meyer-Olkin (KMO) and Bartlett’s test, identified factor structure. Items with loadings < 0.4 or significant cross-loadings were removed [23, 24]. IRT analysis applied a multidimensional graded response model. Items with discrimination parameters (*a*) < 0.75 or difficulty parameters (*b*) outside the − 4 to + 4 range were removed. Estimates were derived using marginal maximum likelihood and the expectation-maximization algorithm [25]. Final item retention was guided by a synthesis of statistical evidence and theoretical coherence.

## Phase 2. Psychometric validation

### Participants and setting

An independent sample of patients with CHD was recruited from the same tertiary hospital between September and November 2022, using identical inclusion and exclusion criteria.

The sample size for Phase 2 was determined based on recommended subject-to-item ratios for confirmatory factor analysis (CFA), with a minimum ratio of 20 participants per item [21]. Given the 12-item structure of the MS-HBC and 20% potential non-response or invalid data rate, the required sample size was at least 300 participants.

### Data collection

Participants completed the finalized MS-HBC alongside the criterion and concurrent measures.

#### Socio-demographics

Basic socio-demographic information, including age, gender, and highest education level, was collected to characterize the study sample.

#### General health motivation

General health motivation was assessed using the relevant subscale of the Motivation to Change Lifestyle and Health Behaviors for Dementia Risk Reduction Scale [15]. This subscale comprises four items rated on a 5-point Likert scale (1 = strongly disagree to 5 = strongly agree) and assesses individuals’ motivation to engage in health-promoting behaviors for long-term health benefits. Higher scores indicate greater motivation. The Chinese version demonstrated acceptable reliability (Cronbach’s α = 0.768; test–retest reliability = 0.892) [26]. This measure was selected as a criterion instrument because it captures trans-situational health motivation, which was hypothesized to represent the foundational drive underlying the disease-specific motivation assessed by the MS-HBC.

#### Motivation to change specific health behaviors

Overall motivation to adopt healthy behaviors was measured using a VAS with the question: “How motivated are you to adopt healthy behaviors?”, rated from 0 (very unmotivated) to 10 (very motivated). The VAS has demonstrated sensitivity in previous studies on health behavior change [11]. It was included as a criterion measure because it provides a direct and global assessment of motivational intensity, serving as a pragmatic benchmark against which the multi-item MS-HBC can be compared.

#### Self-efficacy

Self-efficacy was assessed using the 4-item Short Self-Efficacy for Exercise (SSEE) scale, which measures confidence in overcoming exercise-related barriers on a 5-point Likert scale ranging from 1 (no confidence) to 5 (very confident). It demonstrated good internal consistency (Cronbach’s α = 0.86) in stroke survivors [27]. The SSEE was included to evaluate concurrent validity because self-efficacy is theoretically related to motivation in behavior change models and reflects individuals’ confidence in performing health behaviors [28, 29]. Although the SSEE specifically assesses exercise-related self-efficacy and does not fully encompass broader motivational constructs, exercise represents an important component of lifestyle modification in patients with CHD.

#### Outcome expectation

Outcome expectations were measured using the 5-item Short Outcome Expectation for Exercise (SOEE) scale. Items are rated on a 5-point Likert scale from strongly disagree to strongly agree, with higher scores indicating more positive perceived benefits of exercise. The scale demonstrated strong internal consistency (Cronbach’s α = 0.90) in stroke survivors [27]. It was selected to assess concurrent validity based on theoretical models positing that positive outcome expectations enhance motivation for behavior change [30].

### Data analysis

Statistical analyses were conducted using SPSS version 26.0 and AMOS version 24.0 (IBM Corp., Armonk, NY, USA).

#### Reliability

Internal consistency was evaluated using Cronbach’s α, with values exceeding 0.70 considered acceptable. Test-retest reliability was assessed in a randomly selected subsample of 10% of the participants who completed the MS-HBC again after a two-week interval [31]. Intraclass correlation coefficients (ICCs) above 0.70 were interpreted as indicating satisfactory stability [32].

#### Content validity

Content validity was established during Phase 1 using expert ratings from the Delphi process. I-CVI and scale-level content validity index (S-CVI/Ave) were calculated, with values above 0.78 for I-CVI and above 0.90 for S-CVI/Ave considered acceptable [33].

#### Criterion and concurrent validity

Criterion validity was assessed by correlating MS-HBC scores with two direct measures of motivation: the General Health Motivation subscale and VAS scores. Concurrent validity was examined through correlations with measures of related constructs: self-efficacy (SSEE) and outcome expectations (SOEE). Pearson’s correlation coefficients were used for all analyses.

#### Construct validity

Construct validity was evaluated using CFA within a structural equation modeling framework. Maximum likelihood estimation was applied to assess model fit using absolute fit indices (χ²/df < 5, RMSEA < 0.08, GFI > 0.90), incremental fit indices (NFI > 0.90, CFI > 0.90, TLI > 0.90), and a parsimony fit index (PGFI > 0.50) [34]. Model modifications were considered only when theoretically justified and supported by modification indices.

### Ethical considerations

This study was conducted in accordance with the principles of the Declaration of Helsinki. Ethical approval was obtained from the institutional review boards of the affiliated university (approval No. 2015SY45) and the participating hospital (approval No. 2015030). Written informed consent was obtained from all participants.

## Results

### Phase 1 scale development

#### Sample characteristics

A total of 749 patients participated in this phase (71.6% male; mean age 60.1 ± 9.3 years; 55.2% high school graduates).

#### Item selection

All items except Item 1 (SD = 0.898) met the CTT and IRT criteria (Table 1).

**Table 1:** Item Analysis of the 18-item MS-HBC

No.	SD	CR	α if deleted	CITC	a	b1	b2	b3	b4
item 1	0.898	13.939	0.938	0.735	2.63	-2.31	-1.73	-1.13	-0.15
item 2^*^	1.268	16.451	0.937	0.613	1.35	-2.01	-1.41	-0.65	0.13
item 3	1.076	16.331	0.938	0.740	3.20	-1.80	-1.30	-0.19	0.60
item 4	0.907	16.123	0.937	0.835	4.15	-2.05	-1.47	-0.92	-0.10
item 5	0.982	17.757	0.936	0.858	4.65	-1.86	-1.30	-0.77	-0.08
item 6	1.094	19.009	0.937	0.812	4.36	-1.67	-1.19	-0.18	0.49
item 7	1.088	19.591	0.936	0.832	4.94	-1.65	-1.21	-0.17	0.47
item 8	1.119	20.167	0.935	0.855	4.17	-1.51	-1.04	-0.65	-0.01
item 9	1.165	22.257	0.934	0.845	3.61	-1.42	-1.03	-0.53	0.19
item 10	1.618	34.876	0.940	0.786	5.08	0.11	0.42	0.80	1.06
item 11	1.500	19.636	0.942	0.788	6.73	0.46	0.66	0.95	1.28
item 12	1.120	11.397	0.942	0.664	3.42	0.74	1.12	1.54	1.99
item 13	1.545	19.166	0.942	0.731	3.61	0.43	0.61	0.88	1.35
item 14	1.247	22.625	0.934	0.293	1.14	-2.37	-1.88	-1.35	-0.39
item 15	1.539	42.830	0.934	0.985	13.69	-0.73	-0.24	0.23	0.62
item 16	1.540	43.024	0.934	0.985	13.56	-0.66	-0.22	0.29	0.69
item 17	1.540	45.572	0.934	0.981	13.06	-0.60	-0.27	0.30	0.76
item 18	1.553	42.372	0.934	0.973	11.03	-0.71	-0.34	0.13	0.54

α if deleted = the overall Cronbach’s α when the item is deleted, criteria: the overall Cronbach’s α decreases; a = discrimination parameter, criteria > 0.75; b = difficulty parameter, and the four b values represent the difficulty threshold between options, criteria: [-4, + 4]

*CITC* Corrected item-total correlation, criteria > 0.3, *CR* Critical ratio, criteria > 3, *SD* Standard deviation, criteria > 0.9

*reverse item

### Exploratory factor analysis

EFA was conducted on the 18-item MS-HBC (KMO = 0.923; Bartlett’s test of sphericity, *p* < 0.001), initially extracting three factors that explained 79.75% of the total variance. To optimize the factor structure, six items (Items 2, 8, 9, 14, 1, and 5) were sequentially removed due to substantial cross-loadings, conceptual overlap, or inconsistency with the predefined theoretical dimensions. These refinements improved the clarity and parsimony of the final scale (see Additional file 2 for detailed rationale).

The final 12-item model retained four items per factor, demonstrated a clear three-factor structure aligned with the theoretical framework, and accounted for 86.04% of the cumulative variance (Table 2). The three factors corresponded to the predefined theoretical dimensions:

- Health Motivation Tendencies: Desire to stay healthy by changing unhealthy behaviors or adopting healthy behaviors, driven by cognitive (e.g., knowledge, beliefs) or external factors (e.g., social pressure, perceived health threats), reflecting beliefs in the value of healthy behaviors and willingness to choose them (Contemplation stage).
- Planning Motivation: Effort invested in planning the adoption of healthy behaviors, driven by the desire to be healthy, reflecting preparation for action (transition from Contemplation to Action).
- Action Motivation: Actual adoption of healthy behaviors, indicating maximal motivation, reflecting implementation of specific behaviors (Action stage).

**Table 2:** Factor loadings of the final MS-HBC (12 items)

Items	F1	F2	F3
Item 3 Perceived importance of adopting healthy behaviors for CHD	0.972		
Item 6 Perceived importance of adopting healthy behaviors after illness	0.968		
Item 7 Perceived importance of adopting healthy behaviors after treatment	0.952		
Item 4 Perceived importance of adopting healthy behaviors in daily life	0.707		
Item 11 Level of initiative in seeking relevant information		0.905	
Item 12 Level of initiative in discussing relevant topics with healthcare professionals		0.871	
Item 13 Level of initiative in discussing relevant topics with others		0.856	
Item 10 Level of seriousness in developing a healthy behavior plan		0.812	
Item 16 Level of completion of health behavior plans			0.990
Item 15 Frequency of recent engagement in health behavior initiatives			0.987
Item 17 Level of prioritization of healthy behaviors in daily life			0.983
Item 18 Level of commitment to adopting healthy behaviors			0.974

*CHD* Coronary Heart Disease, *F1* Health Motivation Tendencies, *F20* Planning Motivation, *F3* Action Motivation, *KMO* Kaiser-Meyer-Olkin. Factor loadings below 0.40 are suppressed for clarity. Items are presented in abbreviated form. Full item wording is provided in Additional file 3

## Phase 2. Psychometric evaluation

### Sample characteristics

In phase two, 347 patients were recruited, with the majority being male (*n* = 237, 68.3%). The mean age was 61.3 ± 9.4 years, and over half of the participants were high school graduates (*n* = 219, 63.2%).

### Reliability

The scale demonstrated excellent internal consistency and test-retest reliability. Detailed coefficients, including Cronbach’s α and intraclass correlation coefficients (ICCs), are presented in Table 3.

**Table 3:** Internal consistency and test-retest reliability of the MS-HBC

Scale/Subscale	Cronbach’s α	ICC	95% CI for ICC
MS-HBC (Overall)	0.906	0.981	[0.962, 0.990]
Health Motivation Tendencies	0.933	0.834	[0.696, 0.913]
Planning Motivation	0.887	0.875	[0.766, 0.935]
Action Motivation	0.993	0.894	[0.801, 0.945]

*ICC* Intraclass Correlation Coefficient, *MS-HBC* Motivation Scale for Health Behavior Change, *95% CI* 95% Confidence Interval

### Validity

#### Content validity

All items achieved excellent I-CVIs (0.93–1.00) and an S-CVI/Ave of 0.995.

#### Construct validity

The initial CFA for the 12-item, three-factor model showed an acceptable fit on most indices except for the RMSEA.

**Fig. 1:**
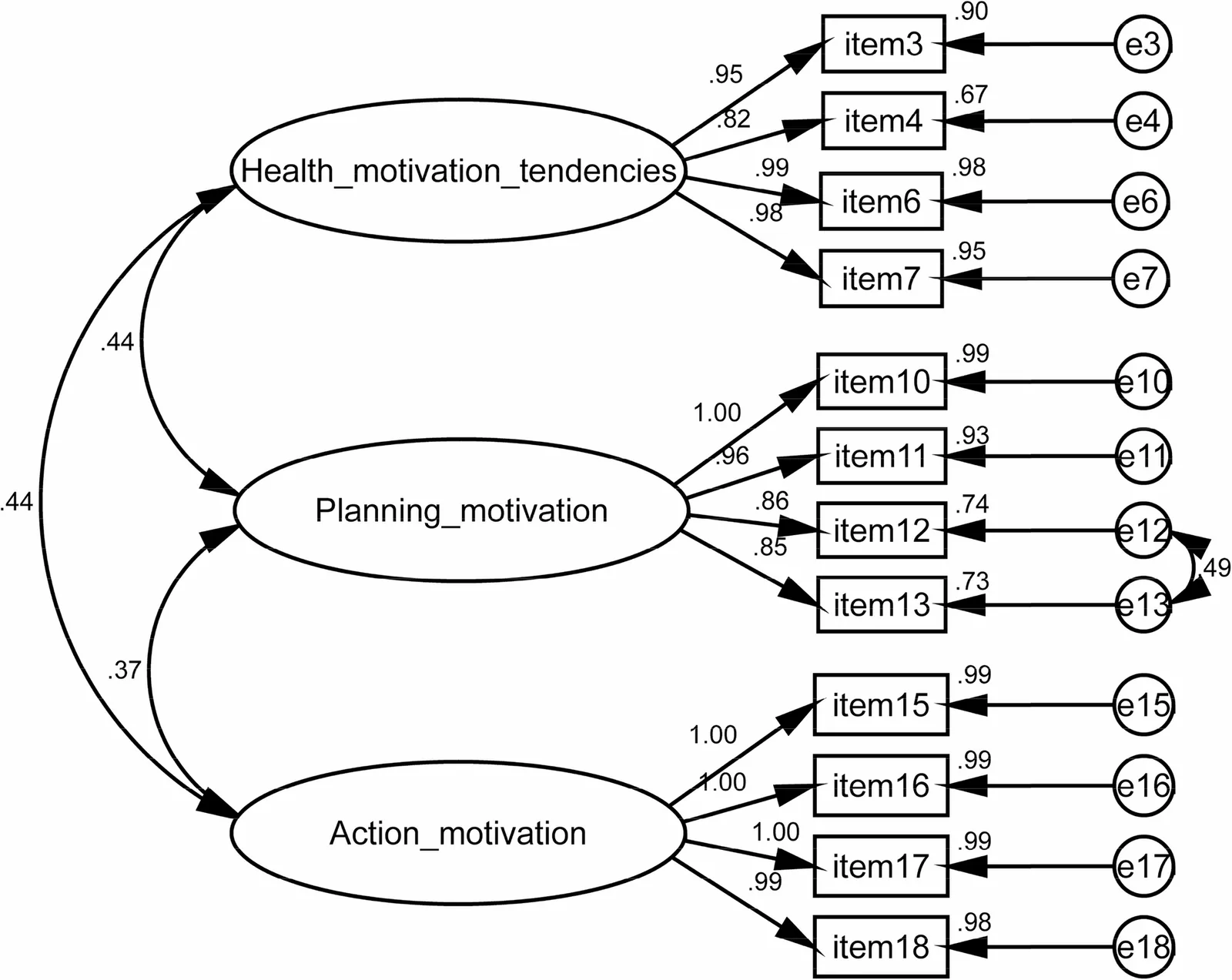
The standardized model diagrams for the modified model of the MS-HBC. CFA was conducted using structural equation modeling. Oval circles represent latent factors; rectangles represent observed variables; circles represent residual error for each observed variable. Numbers close to the 2-headed arrows are correlation coefficients; numbers near the 1-headed arrows between latent factors and observed variables are factor loading coefficients; numbers near the 1-headed arrows between residual errors and observed variables are the variance of observed variables explained by latent factors. Loadings were completely standardized solutions and are all statistically significant

To improve model fit, a theoretically justified modification was applied. The largest modification index suggested a residual correlation between items 12 and 13. This modification was defensible because both items explicitly measure the “degree of initiative” in discussing health plans (with healthcare professionals and social networks, respectively), indicating a shared method variance beyond the latent planning motivation construct. This adjustment resulted in a significantly improved and excellent model fit (Table 4). The standardized structure of this final model is depicted in Fig. 1.

**Table 4:** Fit indices for initial and modified models of the MS-HBC

Model	χ²/df	RMSEA	GFI	NFI	CFI	TLI	PGFI
Criteria	< 5	< 0.08	> 0.90	> 0.90	> 0.90	> 0.90	> 0.50
Initial Model	4.024	0.093	0.915	0.976	0.982	0.977	0.599
Modified Model	2.357	0.063	0.946	0.986	0.992	0.990	0.606

#### Criterion validity

The MS-HBC total score showed a moderate to strong correlation between the total score of MS-HB and both the general health motivation subscale and the VAS (Table 5).

**Table 5:** Pearson correlations between MS-HBC scores and criterion measures

Scale/Subscale	General Motivation Subscale		Visual Analog Scale	
	*r*	95% CI	*r*	95% CI
MS-HBC (Overall)	0.469^**^	[0.388, 0.549]	0.751^**^	[0.703, 0.792]
Health Motivation Tendencies	0.381^**^	[0.281, 0.468]	0.741^**^	[0.672, 0.803]
Planning Motivation	0.385^**^	[0.296, 0.471]	0.367^**^	[0.296, 0.425]
Action Motivation	0.340^**^	[0.246, 0.436]	0.671^**^	[0.607, 0.726]

*MS-HBC* Motivation Scale for Health Behavior Change, *95% CI* 95% Confidence Interval

**p < 0.01; *r* = correlation coefficient

#### Concurrent validity

The MS-HBC total score also showed moderate correlations with scores on the self-efficacy and outcome expectation scales (Table 6).

**Table 6:** Pearson correlations between MS-HBC scores and concurrent measures

Scale/Subscale	Self-Efficacy Scale		Outcome Expectation Scale	
	*r*	95% CI	*r*	95% CI
MS-HBC (Overall)	0.505^**^	[0.416, 0.588]	0.595^**^	[0.524, 0.661]
Health Motivation Tendencies	0.422^**^	[0.325, 0.515]	0.579^**^	[0.470, 0.674]
Planning Motivation	0.303^**^	[0.209, 0.392]	0.317^**^	[0.252, 0.377]
Action Motivation	0.454^**^	[0.358, 0.553]	0.515^**^	[0.439, 0.584]

*MS-HBC* Motivation Scale for Health Behavior Change, *95% CI* 95% Confidence Interval

**p < 0.01; *r* = correlation coefficient

## Discussion

This study developed and validated the 12-item MS-HBC, a novel instrument grounded in the disease-specific CAM model for measuring motivation intensity in patients with CHD. The scale demonstrated promising psychometric properties, including a substantial proportion of variance (86.04%), strong internal consistency (Cronbach’s α = 0.906 for the total scale; ICCs > 0.83), and acceptable-to-good evidence of validity. The MS-HBC contributes to addressing an important gap by providing a theoretically grounded and practically applicable tool tailored to patients with CHD.

The construct validity of the MS-HBC appears to be supported by its theoretical foundation and systematic development process. Grounding the scale in the CAM model helps maintain a clear conceptual distinction between motivation (pre-action drive) and volition (post-action maintenance). This theoretical clarity was empirically supported through an iterative process of factor analysis. The exploratory phase refined the initial 18-item pool into a 12-item model. The removal of items with cross-loadings (e.g., Items 8 and 9 on behavioral choice) enhanced the conceptual coherence of the dimensions, particularly by aligning “Health Motivation Tendencies” more closely with value judgment. The subsequent confirmatory analysis confirmed the three-factor structure. An improved model fit was achieved after allowing a single, theoretically justified residual correlation between items 12 and 13 [35], supporting the structural and theoretical consistency of the final scale. However, as this modification was data-driven and not cross-validated in an independent sample, the stability of the final model should be interpreted with caution.

One advantage of the MS-HBC is its parsimony. Despite its brevity, the scale demonstrated a clear and well-defined factor structure with a substantial proportion of variance accounted for by the extracted dimensions, suggesting good efficiency and practical utility. While more comprehensive scales may capture greater detail, their application in clinical settings is often limited by respondent burden. The MS-HBC, requiring only about two minutes to complete, offers a balance between psychometric adequacy and practical feasibility. This efficiency may facilitate its integration into routine clinical workflows and reduce participant burden in research settings, thereby enhancing feasibility in larger-scale studies.

Notably, the extremely high internal consistency and factor loadings observed in the Action Motivation subscale indicate strong internal coherence, but may also suggest a relatively narrow construct range or potential item redundancy. In the present study, this pattern may also be influenced by the hospital-based sample, where variability in action-related behaviors may be relatively constrained. Further research is warranted to examine construct breadth and refine item content if necessary.

Beyond its efficiency, the MS-HBC may provide useful insights for personalizing patient care. Its distinct subscale scores allow clinicians to move beyond a generic assessment of “low motivation” and instead identify specific motivational profiles. For instance, a patient with high Health Motivation Tendencies but low Planning Motivation may understand the importance of change yet struggle to formulate a concrete plan, thereby benefiting more from structured, clinician-facilitated goal-setting interventions (e.g., action planning) than from general motivational counseling. This diagnostic specificity enables targeted, resource-efficient psychological or behavioral interventions tailored to individual needs.

The MS-HBC occupies a distinct and complementary position in the landscape of motivation assessment. Unlike instruments grounded in Self-Determination Theory, which powerfully evaluate the quality or underlying reasons for motivation (e.g., autonomous vs. controlled) [14], the MS-HBC is purpose-built to quantify the intensity of the drive that bridges intention and action. This focus does not contradict existing theories but rather completes the picture; knowing why a patient is motivated is profoundly enriched by also knowing how strongly that motivation is felt. Future research that integrates these complementary perspectives, assessing both motivational quality and intensity, may contribute to the development of more comprehensive models of health behavior change.

The pattern of correlations with criterion measures provides additional insight into the aspects of motivation captured by the MS-HBC. The stronger correlation with the VAS, which is a direct and global measure of motivational intensity, than with the general health motivation subscale supports the MS-HBC as a measure of perceived motivational intensity, rather than broader health concerns. Furthermore, the moderate-to-strong correlations with self-efficacy and outcome expectations align with established behavioral theories [28–30], confirming that motivation measured by the MS-HBC operates in concert with these well-known drivers of health behavior change.

Several limitations should be considered. First, the initial item pool was primarily theory-driven and derived from existing instruments rather than in-depth qualitative interviews with patients with CHD, which may have limited the inclusion of patient-generated perspectives. Future qualitative studies are warranted to further refine and enrich item content. Second, participants were recruited from a single tertiary hospital during the COVID-19 pandemic, which may limit the generalizability of the findings. In addition, as data were collected in a hospital setting, patients’ motivation levels may have been influenced by the clinical context. Third, regarding measurement, the use of an exercise-specific self-efficacy scale may not fully capture broader aspects of health behavior motivation. Moreover, as participants were asked to reflect on general lifestyle behaviors, responses may have been influenced by variability in how these behaviors were interpreted. Fourth, the Action Motivation subscale demonstrated extremely high internal consistency and factor loadings, which may indicate a relatively narrow construct range or potential item redundancy. Further research is needed to examine and refine this dimension. Finally, the modification applied during confirmatory factor analysis was data-driven and was not cross-validated in an independent sample, which may affect the stability of the final model. Future studies are needed to replicate and validate the factor structure in different samples.

## Conclusions

The MS-HBC is a brief and psychometrically sound instrument for assessing motivation intensity in patients with CHD. Grounded in a clear theoretical framework and supported by initial validation, it may be useful for assessing motivation and informing theory-based behavior change interventions in both research and clinical contexts. Future studies should further validate the MS-HBC in diverse populations and settings to enhance its generalizability. In addition, replication of the factor structure in independent samples is warranted.

## Supplementary Information

Supplementary Material 1.

Supplementary Material 2.

Supplementary Material 3.

## Data Availability

The anonymized dataset supporting the findings of this study has been deposited in Mendeley Data and is publicly available under DOI: 10.17632/m7k8638rmc.1 in Excel format.

## Abbreviations

CAM: Contemplation-Action-Maintenance model
CFA: Confirmatory Factor Analysis
CHD: Coronary Heart Disease
CITC: Corrected Item-Total Correlation
CR: Critical Ratio
CTT: Classical Test Theory
EFA: Exploratory Factor Analysis
ICC: Intraclass Correlation Coefficients
I-CVI: Item-level Content Validity Index
IRT: Item Response Theory
KMO: Kaiser-Meyer-Olkin
MS-HBC: Motivation Scale for Health Behavior Change
SD: Standard Deviation
SOEE: Short Outcome Expectation for Exercise scale
SSEE: Short Self-Efficacy for Exercise scale
VAS: Visual Analogue Scale
