# Supplementary material for: Development and validation of a motivation scale for health behavior change in patients with coronary heart disease: a cross-sectional study
Source: BMC Psychol. 2026 Jul 3;14:1381. doi: 10.1186/s40359-026-05114-z (PMC13602562; doi:10.1186/s40359-026-05114-z)
Supplement: Supplementary file 1 [file 40359_2026_5114_MOESM1_ESM.docx]

### Additional file 2: The Reasons for the Item Deletion

**Table 1 Exploratory Factor Analysis for the 18-item MS-HBC**

| **Item** | **F1** | **F2** | **F3** |
| --- | --- | --- | --- |
| Item 3 Perceived importance of adopting healthy behaviors for CHD | 0.932 |  |  |
| Item 6 Perceived importance of adopting healthy behaviors after illness | 0.927 |  |  |
| Item 7 Perceived importance of adopting healthy behaviors after treatment | 0.914 |  |  |
| Item 4 Perceived importance of adopting healthy behaviors in daily life | 0.819 |  |  |
| Item 5 Concern about benefits of healthy behaviors | 0.778 |  |  |
| Item 1 Perceived threat of unhealthy behaviors to health | 0.771 |  |  |
| Item 8 Willingness to adopt healthy behaviors | **0.616** |  | **-0.424** |
| Item 9 Urgency to adopt healthy behaviors | **0.591** |  | **-0.446** |
| Item 11 Level of initiative in seeking relevant information |  | 0.895 |  |
| Item 12 Level of initiative in discussing relevant topics with healthcare professionals |  | 0.854 |  |
| Item 13 Level of initiative in discussing relevant topics with others |  | 0.848 |  |
| Item 10 Level of seriousness in developing a healthy behavior plan |  | 0.824 |  |
| Item 16 Level of completion of health behavior plans |  |  | -0.978 |
| Item 15 Frequency of recent engagement in health behavior initiatives |  |  | -0.974 |
| Item 17 Level of prioritization of healthy behaviors in daily life |  |  | -0.969 |
| Item 18 Level of commitment to adopting healthy behaviors |  |  | -0.962 |
| Item 14 Determination to execute healthy behavior plan | **0.437** |  | **-0.587** |
| Item 2 Level of satisfaction with current health behavior |  |  | **-0.509** |
| **Initial eigenvalue** | 9.766 | 2.613 | 1.976 |
| **Rotation sums of squared loadings** | 8.018 | 3.839 | 7.667 |
| **Cumulative variance explained (%)** | 79.75 | | |
| **KMO** | 0.923 | | |
| **Bartlett test of sphericity** | <0.001 | | |

Note: CHD = Coronary Heart Disease; F1 = Health Motivation Tendencies, F2 = Planning Motivation, F3 = Action Motivation; KMO= Kaiser-Meyer-Olkin. Factor loadings below 0.40 are suppressed for clarity.

Item 2, *“The degree of my satisfaction with current health behavior is:”,* was deleted due to theoretical misfit. Although intended to measure Health Motivation Tendencies, it loaded alongside items defining Action Motivation, indicating it did not measure the intended construct. Its removal increased the cumulative variance to 81.49%.

Items 8, 9 were removed to improve dimension homogeneity. Item 8, “*The intensity of my willingness to adopt healthy behaviors is:*”, and item 9, “*The degree of my urgency to adopt healthy behaviors is:*”, were used to measure willingness and urgency to adopt behaviors , cross-loaded on multiple factors and conceptually differed from the core theme of the dimension (belief in behavior value). Their deletion refined the dimension definition and increased variance to 79.88%.

Item 14 was deleted due to construct misinterpretation. Item 14, *“After developing a healthy behavior plan, my determination to execute it is:”,* measured determination to execute a plan (aligning more with Action Motivation) rather than the initiative to make a plan (the core of Planning Motivation). Its removal increased variance to 79.81%.

Items 1 and 5 were eliminated due to conceptual redundancy. Both item 1 (*The degree of threat to my health from unhealthy behaviors is:*) and item 5 (*The degree of my concern about the benefits of healthy behaviors is:*) were similar to item 4 (*The importance of healthy behaviors in my daily life is:*), failing to provide unique information. Their sequential removal increased the cumulative variance to 81.00% and 79.95%, respectively.

This iterative process resulted in a final 12-item scale with a clear three-factor structure, accounting for 86.04% of the total variance.
