# Supplementary material for: Development and validation of a motivation scale for health behavior change in patients with coronary heart disease: a cross-sectional study
Source: BMC Psychol. 2026 Jul 3;14:1381. doi: 10.1186/s40359-026-05114-z (PMC13602562; doi:10.1186/s40359-026-05114-z)
Supplement: Supplementary file 2 [file 40359_2026_5114_MOESM2_ESM.docx]

**Additional file 1: The 14 Existing Scales Involved in Building the Item Pool**

**Name of the 14 existing scales**

1. The quitting smoking motivation scale (Liyan Zhao 2012) ^1^
2. The Health Motivation Scale in Healthy Eating (Xu2009) ^2^
3. The Health Motivation Scale in Physical Activities (Xu2009) ^2^
4. General motivation dimension in the Motivation to Change Lifestyle and Health Behaviours for Dementia Risk Reduction Scale (Kim2014) ^3^
5. The Self-Concept and Motivation to Exercise Scale (Wilson2002 ) ^4^
6. The Q-MAT Smoking Cessation Motivation Questionnaire (Aubin2005) ^5^
7. Health Motivation dimension in the Health Belief Model scale (Champion2010) ^6^
8. A single-item motivation scale for quitting smoking (Beard2012) ^7^
9. The Readiness and Motivation to Quit Smoking Scale (Crittenden1994) ^8^
10. The Arabic Readiness to Stop Smoking Questionnaire (Haddad2001) ^9^
11. The Motivation to Stop Scale (Kotz2013) ^10^
12. The Readiness to Change Exercise Questionnaire (Kheawwan2016) ^11^
13. The State Motivation Scales for Alcohol Control (Robinson2016) ^12^
14. The Motivational Thought Frequency Scales (Robinson2016) ^12^

**References**

1.	Zhao LY. The effect of health motivation on college students' smoking cessation behavior. Master thesis. Sichuan Normal University; 2012. (in Chinese)

2.	Xu X. Health motivation in health behavior: Its theory and application. Accessed 2023-5-31, <https://digitalscholarship.unlv.edu/cgi/viewcontent.cgi?httpsredir=1&article=1044&context=thesesdissertations>

3.	Kim S, Sargent-Cox K, Cherbuin N, Anstey KJ. Development of the motivation to change lifestyle and health behaviours for dementia risk reduction scale. Dement Geriatr Cogn Dis Extra. May 2014;4(2):172-83. doi:10.1159/000362228

4.	Wilson DK, Friend R, Teasley N, Green S, Reaves IL, Sica DA. Motivational versus social cognitive interventions for promoting fruit and vegetable intake and physical activity in African American adolescents. Annals of Behavioral Medicine. Fal 2002;24(4):310-319.

5.	Aubin H-J, Lagrue G, Legeron P, et al. Smoking cessation motivation questionnaire (Q-MAT) : Construction and validation. Alcoologie et addictologie. 2005;27(2):19s-25s.

6.	Champion V. The relationship of breast self-examination to health belief model variables. Research in Nursing & Health. 2010;10(6):375-382.

7.	Beard E, Aveyard P, Brown J, West R. Assessing the association between the use of NRT for smoking reduction and attempts to quit smoking using propensity score matching. Article. Drug and Alcohol Dependence. 2012;126(3):354-361. doi:10.1016/j.drugalcdep.2012.05.039

8.	Crittenden KS, Manfredi C, Lacey L, Warnecke R, Parsons J. Measuring readiness and motivation to quit smoking among women in public health clinics. Addict Behav. Sep-Oct 1994;19(5):497-507. doi:10.1016/0306-4603(94)90005-1

9.	Haddad LG, Hoeman SP. Development of the Arabic language readiness to stop smoking questionnaire A-RSSQ. Journal of Nursing Scholarship. 2001 2001;33(4):355-359. doi:10.1111/j.1547-5069.2001.00355.x

10.	Kotz D, Brown J, West R. Predictive validity of the Motivation To Stop Scale (MTSS): a single-item measure of motivation to stop smoking. Drug Alcohol Depend. Feb 1 2013;128(1-2):15-9. doi:10.1016/j.drugalcdep.2012.07.012

11.	Kheawwan P, Chaiyawat W, Aungsuroch Y, Wu YWB. Patient Readiness to Exercise After Cardiac Surgery: Development of the Readiness to Change Exercise Questionnaire. Article. The Journal of cardiovascular nursing. 2016;31(2):186-193. doi:10.1097/JCN.0000000000000221

12.	Robinson N, Kavanagh D, Connor J, May J, Andrade J. Assessment of motivation to control alcohol use: The motivational thought frequency and state motivation scales for alcohol control. Addict Behav. Aug 2016;59:1-6. doi:10.1016/j.addbeh.2016.02.038
