# Supplementary material for: Development and validation of a motivation scale for health behavior change in patients with coronary heart disease: a cross-sectional study
Source: BMC Psychol. 2026 Jul 3;14:1381. doi: 10.1186/s40359-026-05114-z (PMC13602562; doi:10.1186/s40359-026-05114-z)
Supplement: Supplementary file 3 [file 40359_2026_5114_MOESM3_ESM.docx]

**Additional file 3**

Note: Psychometric validation in this study was conducted using the Chinese version. The English version of the MS-HBC is provided for reference purposes only to facilitate international readability. Users intending to apply the English version should conduct appropriate validation in the target population before use.

**Chinese Version (Validated)**

**健康行为改变动机量表**

指导语：低盐低脂饮食、每周至少150分钟的适度运动以及戒烟对减少冠心病的并发症，提高冠心病患者的生活质量有重要作用。相反，不健康饮食、不规律运动和吸烟的不健康行为会增加冠心病患者再次发病率和死亡率。关于采取这些行为的动机，请您阅读以下题目，并根据实际情况进行5级评分，1分为程度最低，5分为最高，请在相应的分数上画“√”。

| **条目** | **极低** | **较低** | **一般** | **较高** | **极高** |
| --- | --- | --- | --- | --- | --- |
| ***健康动机趋势*** | | | | | |
| 1. 对于我的病来说，我认为采取健康行为的重要程度： |  |  |  |  |  |
| 2. 我认为采取健康行为在日常生活中的重要程度： |  |  |  |  |  |
| 3. 得了病之后再来采取健康行为，我认为意义大小： |  |  |  |  |  |
| 4. 如果已经做了手术或者吃了治疗药物，我认为采取健康行为的重要程度： |  |  |  |  |  |
| ***计划动机*** | | | | | |
| 5. 我制定健康行为计划的认真程度： |  |  |  |  |  |
| 6. 为了制定出适合自己的健康行为计划，我自行查阅书籍或网络上相关信息的主动程度： |  |  |  |  |  |
| 7. 为了制定出适合自己的健康行为计划，我与医生或护士讨论的主动程度： |  |  |  |  |  |
| 8. 为了制定出适合自己的健康行为计划，我与病友或家人朋友等谈论改变健康行为相关话题的主动程度： |  |  |  |  |  |
| ***行动动机*** | | | | | |
| 9. 近期我开始有意识地执行健康行为计划的频次： |  |  |  |  |  |
| 10. 我每次执行健康行为计划的完成程度： |  |  |  |  |  |
| 11. 在每天做的事情中，我采取健康行为的优先程度： |  |  |  |  |  |
| 12. 每次采取健康行为时，我的认真程度： |  |  |  |  |  |

**English Version (Only for reference)**

**The motivation scale for health behaviour change（MS-HBC）**

*Guidance: Lifestyle behaviors such as a low-salt and low-fat diet, engaging in at least 150 minutes of moderate physical activity per week, smoking cessation, and adherence to prescribed medication play an important role in reducing complications and improving quality of life in patients with coronary heart disease (CHD). In contrast, unhealthy behaviors may increase the risk of disease progression and adverse outcomes.*

*When responding to the following items, please consider your current situation regarding these behaviors. For each statement, indicate the extent to which it reflects your current condition by selecting one option on a 5-point scale (1 = extremely low, 5 = extremely high).*

| **Items** | **extremely low** | **low** | **neutral** | **high** | **extremely high** |
| --- | --- | --- | --- | --- | --- |
| *Health Motivation Tendencies* | | | | | |
| 1. The importance of adopting healthy behaviors for my condition is: |  |  |  |  |  |
| 2. The importance of adopting healthy behaviors in my daily life is: |  |  |  |  |  |
| 3. The importance of adopting healthy behaviors after being diagnosed with CHD is: |  |  |  |  |  |
| 4. The importance of adopting healthy behaviors after receiving treatment is: |  |  |  |  |  |
| *Planning Motivation* | | | | | |
| 5. My level of commitment to developing a healthy lifestyle plan is: |  |  |  |  |  |
| 6. My initiative to seek relevant information (e.g., books or the internet) for developing a suitable plan is: |  |  |  |  |  |
| 7. My initiative to discuss relevant topics with healthcare professionals is: |  |  |  |  |  |
| 8. My initiative to discuss relevant topics with others (e.g., family, peers) is: |  |  |  |  |  |
| *Action Motivation* | | | | | |
| 9. My frequency of consciously implementing healthy behavior plans is: |  |  |  |  |  |
| 10. My level of completion of health behavior plans each time is: |  |  |  |  |  |
| 11. The priority I give to adopting healthy behaviors in daily life is: |  |  |  |  |  |
| 12. My level of commitment when carrying out healthy behaviors is: |  |  |  |  |  |
